# Cripping the Transition to Practice: A Longitudinal Qualitative Analysis of Disabled Doctors’ First Year Experiences

**DOI:** 10.1007/s40670-026-02873-7

**Published:** 2026-09-05

**Authors:** Megan E.L. Brown, Anna Harvey Bluemel, Oliver Hope, Matthew Byrne, Gabrielle Finn, Neera R. Jain

**Affiliations:** 1 School of Medicine, Newcastle University, Newcastle, UK; 2 School of Medicine, University of Sunderland, Sunderland, UK; 3 Nuffield Department of Surgical Sciences, University of Oxford, Oxford, UK; 4 School of Medical Sciences, Faculty of Biology, Medicine and Health, University of Manchester, Manchester, UK; 5 Centre for Medical and Health Sciences Education, Faculty of Medical and Health Sciences, Waipapa Taumata Rau – The University of Auckland, Auckland, New Zealand

**Keywords:** Disabled, Disability, Transition, Medical education, Longitudinal

## Abstract

**Introduction:**

The transition from medical student to doctor is demanding. Although research has explored preparedness and wellbeing, disabled doctors’ experiences remain underexamined, risking a homogenised understanding of a process that is likely experienced unevenly across learner groups.

**Materials and methods:**

We conducted a longitudinal qualitative study using a phenomenological approach, following six disabled final-year medical students through their first year of postgraduate practice in the UK. All six completed interviews before and shortly after starting work, four completed a third interview, and two completed all four interviews. The 18 interviews were analysed using reflexive thematic analysis, with crip theory informing interpretation.

**Results:**

We constructed four themes: negotiating legitimacy and belonging, living with the fluctuating pace of clinical practice, access work as ongoing labour, and seeking survivable futures. Belonging rose and fell across placements, access arrangements were repeatedly renegotiated, and fluctuations in pace shaped wellbeing and participants’ sense of professional legitimacy. Difficulties did not follow a consistent trajectory of decline. Rather, participants developed strategies for navigating recurring tensions, often at significant personal cost. By the end of the year, some were orienting career planning toward contexts where practice felt sustainable.

**Discussion:**

Transition for disabled doctors was recurrent and context dependent, with changing placements requiring renewed negotiation of disability legitimacy and access. PGME support should reduce the individualised labour of maintaining adjustments across rotations and address how expectations concerning pace and productivity shape supervision and judgements of competence.

**Supplementary Information:**

The online version contains supplementary material available at https://doi.org/10.1007/s40670-026-02873-7.

## Introduction

The transition from medical student to doctor is potentially the most challenging of a medical career, involving psychological, social, and educational adaptation as role, context, relationships, and professional identity [1] change. New doctors face elevated risk of burnout, which can lead to attrition [2] and negatively impact patient care [3]. Though research has explored preparedness for practice [4, 5], and the emotional experience of beginning work [6, 7], little research explores the experiences of disabled doctors during this critical period, risking a homogenised understanding. Recent research by Keenan et al. [8] into UK Autistic doctors’ transition to clinical practice, for example, suggests that transition is experienced unevenly and that professional identity development may involve continuous negotiation rather than a straightforward progression toward confidence and independence for neurodivergent doctors. Keenan’s work questions the sufficiency of current frameworks in conceptualising transition for diverse learners.

Popular frameworks for understanding transition draw on educational, developmental, and social lenses, positioning it as a process of individual adjustment toward preparedness, confidence, and social integration into clinical environments [9]. These dominant conceptualisations of transition matter because they locate responsibility for difficulty within individuals. Scholars have raised parallel concerns within professional identity research, arguing that dominant frameworks have produced normative accounts of identity development that inadequately capture the experiences of marginalised learners [10–12]. When transition is framed primarily as a matter of preparedness or personal resilience, challenges can be interpreted as resulting from individual learner shortcomings, rather than a workplace’s expectations, or broader occupational norms [13]. Within medicine, early clinical practice has traditionally been associated with the ‘hazing’ of new graduates through high workloads and strong cultural expectations around competence and endurance [14]. Transition may also function as a process of socialisation into professional norms [15].

Critical disability studies offer alternative ways to conceptualise transitions. Crip theory spotlights how institutional norms ensnare participants into attempts to embody unreachable standards of being and doing, rendering other ways problematic or exceptional [16]. From this perspective, transition to practice can be understood as a process whereby professional norms are enacted and reinforced, including expectations about pace and productivity. Crip theory also emphasises the potential to shift dominant norms, by productively failing to embody them, a process known as *cripping* [16, 17]. Disabled learners entering clinical practice may therefore encounter additional layers of negotiation as they navigate systems that assume non-disabled performance, with the potential to assimilate to, seek to adapt, or resist these systems.

Research on disability in medical education has documented barriers to career progression, difficulties obtaining adjustments, and variation in institutional disability policies [18–27]. This work establishes that disabled learners and doctors can experience exclusion across undergraduate medical education (UME) and postgraduate medical education (PGME). Much of it examines experiences or institutional practices at a single point in time, however, and cannot show how disability-related experiences develop as doctors move from university into employment and between clinical placements. Existing guidance on the UME to PGME transition identifies disability disclosure, specialty and programme selection, and access to accommodations as areas requiring support [28]. This guidance provides practical recommendations, but prospective empirical evidence about how these processes are experienced across the transition remains limited.

Recent phenomenological research with autistic doctors has shown that transition into clinical practice can involve unpredictability, powerlessness, and continuing negotiation of professional identity [8]. This provides a detailed account of transition for one disability group based on interviews conducted after participants had entered practice. We do not yet know how concerns expressed before work compare with subsequent experiences, or how the same disabled doctor’s experience changes as clinical teams, working practices, and access arrangements change. Research across a range of disability experiences is also required to examine cross-cutting processes of disablement within PGME while retaining differences between impairments and access needs.

This temporal gap affects our understanding of transition difficulties. Without prospective, repeated accounts, difficulties encountered during F1 may be attributed to limited preparedness or the expected pressures of becoming a new doctor when they may, instead, be a product of the interaction between disability and particular clinical environments. Difficulties may also be confirmed, reduced, reinterpreted, or reproduced as doctors move between placements. Following participants from before they begin work allows us to examine these changes within individual accounts and also has the potential to show whether support established at one point is sustained as organisational responsibility and working relationships change.

We therefore conducted a prospective longitudinal phenomenological study of disabled doctors’ transition from UME into UK clinical practice. We examined how disability and access were experienced and negotiated from before doctors qualified, through their first postgraduate year. By following doctors with different disabilities across repeated interviews, we sought to identify disability-related processes occurring across accounts while retaining variation in how participants experienced them. Crip theory informed our interpretation of how professional expectations and PGME arrangements shaped doctors’ experiences. Specifically, we asked:

1. What are the lived experiences of disabled learners as they begin work as doctors in the UK?
2. How do disabled doctors’ experiences change as they move through the first year of work?

## Materials and Methods

### Context

First year UK doctors are provisionally registered with their regulatory body, the General Medical Council (GMC). They must enter a two-year training programme, the Foundation Programme, to work as a doctor^1^. This programme involves six four-month placements across primary and secondary care settings. Satisfactory completion of the first year (Foundation Year 1 or ‘F1’) results in full GMC registration and leads into Foundation Year 2 (F2). New doctors completing F2 are eligible to enter a specialty training programme of their choice. Foundation Programme placements are allocated randomly, according to ranked geographical preferences.

### Positionality

Our team comprises researchers from the Universities of Newcastle, Manchester and Auckland, and the MedEd Collaborative (a group of UK trainee doctors building capacity in clinical education research through practical experience) [29]. Collectively, we have lived and professional experience of disability, UK clinical practice, undergraduate medical education (UME), and PGME. These perspectives shaped our interest in the study, its design, and our sensitivity to issues of workplace culture during analysis.

### Study Design

We selected a phenomenological approach situated within an interpretivist paradigm to engage closely with how participants interpreted and made meaning of their experiences [30]. Our phenomenon of interest was the lived experience of entering clinical practice as a disabled doctor, including how disability and access were interpreted and negotiated across placements. We, therefore, adopted a longitudinal qualitative design, conducting multiple, repeat interviews from completion of UME through the end of F1. This allowed us to revisit earlier expectations and examine how participants interpreted subsequent experiences across placements [31].

We designed our research methods for accessibility (e.g., online interviews), offering flexible options (e.g., longer interviews, email or voice note interviews) and budgeting for access costs (e.g., British Sign Language interpretation) [32]. Whilst some participants required longer interviews, all opted for synchronous 1:1 interviews via Microsoft Teams.

Newcastle University’s Ethics Board granted approval (Ref:45663/2023).

### Recruitment and Participants

We recruited participants via social media (X and LinkedIn) using researchers’ professional profiles and ASME and the MedEd Collaborative’s institutional profiles. Interested final year medical students self-identifying as disabled, neurodivergent, D/deaf, and/or as someone who experiences impairment, chronic illness, or mental health concerns were invited to complete a short form. Two authors (MELB/AHB) provided everyone who met the participation criteria with a participant information sheet and offered the opportunity to ask questions. Participants provided written informed consent prior to enrollment and were offered a £20 voucher per interview in gratitude for their participation.

Recruitment took place May-June 2024, before the 2024 Foundation Programme began, and closed when that cohort commenced F1, allowing prospective follow-up from a common transition point. Six participants joined the study.

The sample was intentionally small to support repeated, in-depth engagement with individual accounts, consistent with the study’s phenomenological approach [30]; it was not intended to represent all disabled doctors’ experiences. Participants were heterogeneous in their disabilities and access needs but shared the phenomenon of entering clinical practice as disabled doctors. Disability type was not treated as a category for comparison. We examined disability-related processes across accounts while retaining differences in how participants experienced and interpreted them. The study was not designed to produce condition-specific conclusions.

Participants described physical and mobility impairments, sensory and auditory-processing disabilities, dyslexia, ADHD, chronic illness and mental health conditions, and several described more than one disability. We report characteristics in aggregate and include attributable information only where required to interpret an account. A participant-level characteristics table is not included as it would combine a small number of potentially identifying features in a sample recruited through public social-media networks and risk breaching confidentiality.

### Data Collection

Two authors (MELB and AHB) conducted all semi-structured interviews, at four points (T1-T4):

1. Prior to commencing F1 (June/July 2024).
2. One-two months after commencing F1 (September/October 2024).
3. Six months after commencing F1 (March 2025).
4. Approaching commencement of F2 (July 2025).

Wherever possible, the same author interviewed participants throughout. Our interview guide (see Supplementary material) was grounded in transition literature and relevant issues for disabled doctors, then tailored using topics raised in each participant’s preceding interview. Following each interview, participants were debriefed by email and invited to the subsequent timepoint interview. At each timepoint, participants who did not respond to the initial invitation received up to three email reminders. Flexible options for participants, including shorter interviews and interviews conducted by email or voice note, remained available but were not taken up. Participation at each timepoint was voluntary, and participants were not required to provide a reason for discontinuing.

We invited participants to select pseudonyms [33] to represent their data. Three opted to do so (two selected names, one selected an adjective “Resourceful”), for the other three we used a random name generator for consistency. All names in the Results are pseudonyms and should not be used to infer participant gender.

Attrition occurred at later time points. All six participants completed T1 and T2, four completed T3, and two completed T4. This produced 18 interviews. Table 1 shows participation at each timepoint.

**Table 1:** Participant numbers for each study interview

Interview number	Number of participants
1	6
2	6
3	4
4	2

### Data Analysis

We conducted a reflexive thematic analysis of our interview data using Braun and Clarke’s approach [34], a methodologically flexible approach to analysis aligned with our overall aim to present a rich interpretation of participant experiences. Crip theory was used as a sensitising lens during theme development and interpretation, it was not a predetermined coding framework.

The interviewing authors (MELB/AHB) produced verbatim transcripts, refining Microsoft Teams auto-transcripts. MELB and OH led the analysis. AHB, GF, and NRJ supported coding at T1 and T2 to enhance team familiarity with the evolving dataset. Following Braun and Clarke’s recursive phases [34], the team familiarised themselves with the data, generated codes, constructed and reviewed candidate themes, defined and named themes, and refined the analysis through writing. Each timepoint was initially coded as one dataset. MELB and OH then compared codes and candidate patterns across timepoints and returned to individual participants’ earlier and later interviews to examine continuity, change, and contradiction. They developed one set of themes representing recurrent processes across the year. Candidate themes and supporting extracts were circulated to the wider team at several stages for reflexive discussion and further revision.

### Reflexivity

We kept reflective memos and reflected together during team meetings. Longitudinal reflective memos were used when conceptualizing themes to facilitate team discussion regarding the data, its evolution through time, and researchers’ interpretations. Smaller groups also discussed their engagement with the data by email and in person, supporting ongoing reflexive dialogue and examination of assumptions.

## Results

The longitudinal dataset comprised 18 interviews: all six participants completed T1 and T2, four completed T3, and two completed T4. Quotation labels identify the interview timepoint. Across four timepoints, participants described experiences that were cyclical rather than linear: belonging rose and fell across placements; access arrangements were repeatedly negotiated; and pace and workload fluctuated in ways that impacted participants’ capacity to cope throughout their first year of practice. We examined change within participants alongside patterns across timepoints, and have been careful not to assume that participants moved through the same stages.

Participants’ early expectations were subsequently confirmed, reduced, or reinterpreted as they entered different clinical environments. There was no common progression towards resolution. Some difficulties eased when participants became familiar with a team or gained greater control over their work, while others emerged or recurred when working patterns, relationships and access arrangements changed. Formal support could reduce uncertainty, but it did not consistently remove the labour required to establish and maintain access.

Although participants described many challenges common to transition into medical practice, including uncertainty, changing responsibilities and workload, their accounts consistently situated these experiences within the additional work of negotiating disability legitimacy, access needs and organisational expectations.

Themes are summarised in Table 2.

**Table 2:** Summary of themes

Theme	Summary, and change within the theme through time
Negotiating legitimacy and belonging	Belonging became more secure within some teams but was disrupted by rotation, visible adjustments (which could lead to resentment and stigma), and negative, judgmental attitudes relating to their competence or entitlement to support.
Living with the fluctuating pace of clinical practice	Participants anticipated workload effects before F1. Later interviews showed that pace interacted with specific environments: collective, fast tasks intensified difficulty, while greater control sometimes reduced it.
Access work as ongoing labour	Preparation for securing accessibility arrangements began before employment, but agreed support did not consistently transfer into work or across placements. Participants had to repeatedly identify those responsible for providing access to support, explain their needs, and follow up with implementation.
Seeking survivable futures	Participants entered F1 with provisional career concerns. Their experiences across their first-year rotations tested and refined their plans, producing more specific judgements about workable environments rather than a common endpoint.

### Negotiating Legitimacy and Belonging

Participants described legitimacy as unstable and continually negotiated, operating at two interconnected levels: professional legitimacy (being seen as a competent new doctor); and disability legitimacy (whether adjustment needs were viewed as justified or excessive). Participants anticipated that visible adaptations could destabilise colleagues’ perceptions of their professional identity, making belonging feel conditional rather than secure.

Early interviews revealed vigilance about how participants were perceived, including concern that disability disclosure might shape first impressions. For Alva, moving repeatedly between unfamiliar teams as a medical student made these decisions harder:

“*I find it very stressful making… a first impression every day with a different team.” (Alva*,* T1).*

They generally did not disclose their disability straight away because people could have *“preconceived ideas”* about what their diagnosis meant, and preferred colleagues to know them first. Frequent rotations between different clinical placements also made it *“impossible”* or “*very difficult”* to ask for adjustments. These experiences as a medical student shaped Alva’s concerns moving into the first year of work. Reassuringly, two months into F1, Alva felt they had *“slotted in quite nicely” (Alva*,* T2)* with the ward team. Asked about disclosure, they described a supportive local culture:

*“I think that I haven’t had any… negative responses*,* which again I think is… part of the culture in the trust being quite good.” (Alva*,* T2).*

This relative security was connected to the relationships and culture of that placement. However, the experience of disclosure varied between participants. Victoria’s later interview at T4 showed how even when well-established as a doctor, after a year of clinical practice, Victoria felt she still had to counter negative assumptions from colleagues arising from her visible disability:

*“I feel like I need to prove that I’m… just as efficient*,* just as able*,* just as a capable doctor as anyone there.” (Victoria*,* T4).*

Across the study, participants made careful decisions about what to tell colleagues about their disability or about negative disability-related experiences. Disclosure was, therefore, rarely a single decision; it shifted across contexts and relationships. For some participants, visible markers of disability shaped these interactions. Elise, who wore hearing aids, described how visibility reduced the need for repeated explanation, but they adapted disclosure of their disability based on context:

*“I think usually if I have my hair up*,* people can see my ears. So it’s sort of an unspoken… if I’m working with someone directly… I’d mention it if necessary.”(Elise*,* T2)*.

Participants also distinguished between disclosing disability-related needs and deciding whether to report poor treatment. Reporting negative experiences involved additional calculations about credibility and the risk of being perceived as complaining. Victoria described how she filtered which negative experiences to report:

*“If I reported every single little thing*,* at some point*,* you would stop caring… I’ve ended up having to filter what was*,* ‘Ugh*,* that’s annoying*,*’ versus ‘Okay*,* that’s really inappropriate.’” (Victoria*,* T1)*.

As the year progressed, legitimacy remained fragile. When adaptations became more visible, participants described a shift from needing to prove professional competence toward having their entitlement to support scrutinised. Resourceful described how peer relationships changed once adjustments became apparent:

*“They began to get very unkind to me… they began to dissect did my health conditions really warrant the level of adaptions that I was getting.” (Resourceful*,* T3)*.

Across interviews, legitimacy and belonging remained contingent on clinical context. Alva’s account in this theme showed that anticipated disability stigma could be reduced within a supportive local culture. Victoria’s accounts shows that vigilance about reporting disability-related poor treatment at T1 remaining alongside a continuing need to prove her competence at T4. Resourceful’s account shows how the visibility of adjustments can introduce new scrutiny of competence and entitlement to support. These accounts do not indicate a common progression from insecurity towards belonging. Rather, they show that professional and disability legitimacy had to be repeatedly established and defended within particular teams. Participants felt more secure in some settings and became vulnerable to scrutiny in others.

### Living with the Fluctuating Pace of Clinical Practice

Participants described clinical practice as demanding a pace that often felt difficult to sustain. Early interviews contained anticipation and planning around how their bodies or cognition might respond to workload, including decisions about working patterns and roles. Before starting F1, Resourceful described the uncertainty involved in being asked to specify adjustments without experience of the job:

*“It’s the unknown unknown of what support I’ll need… of what working shifts will do to me*,* of what working nights will do.” (Resourceful*,* T1).*

Victoria described planning to work less than full time to manage the demands of her healthcare:

*“I’m going to go 80% hours just to give me that extra day off a week because I’m hoping that that will help with managing appointments… it literally is… a full-time job just managing my health.” (Victoria*,* T1)*.

At T1, Maddi was *“very scared”* about starting F1 because of their health. By T2, fast ward rounds were a context in which that concern had been realised. They described the pressure of having to be ‘so on it’ to keep up: “*I find the ward rounds the hardest bit… they move so fast… you have to just be so on it… it can be difficult to just keep up with that pace.” (Maddi*,* T2).*

Alva described transition as involving a sudden expansion of responsibility: *“Suddenly I was expected to… manage all of my life.” (Alva*,* T2)* Thus, pace was experienced also through the accumulation of personal, professional, and organisational demands.

Pace was also a form of social expectation. Keeping up signalled competence, while struggling risked being seen as less capable. However, this did vary across placements. Jenny’s interviews showed how the organisation of work across different placements changed the impact of her disability. At T1, she anticipated taking longer to familiarise herself with terminology but expected eventually to adapt. In a geriatric medicine placement at T3, fast board rounds and ward rounds made this more consequential:

*“They use a lot of terminology… my brain tries to process… and I’m missing out the rest of the information.” (Jenny*,* T3)*.

At T4, Jenny’s experience in Same Day Emergency Care (SDEC) differed again. Conducting consultations at her own pace allowed her to clarify information and reduced the effect of her auditory-processing difficulties:

*“SDEC… is actually less stressful for me… you kind of do your own consultation yourself*,* like using your own pace.” (Jenny*,* T4)*.

Participants moved through cycles of exhaustion and recovery across rotations and through time. Sustainable pace was often temporary, followed by renewed pressure in subsequent rotations. Some participants attempted to manage this through planning or formal adaptations, although these did not always resolve challenges. Maddi found working less-than-full-time helpful, but unpredictable in practice:

*“I’m 80% so that is helping… every day is just so different. Some days are fine*,* and then other days I’m like*,* I can’t do this.” (Maddi*,* T2)*.

Adaptations could also introduce new social tensions linked to legitimacy. Maddi recalled a supervisor asking: *“so what do you do with the other 20%?” (Maddi*,* T2).* Rather than consistently diminishing with experience, the demands of pace persisted, with later interviews highlighting how sustained exposure could lead to exhaustion and extended working time. For Resourceful, peer relationships had deteriorated after colleagues questioned their adjustments. Following difficult responses when work was handed over, Resourceful stopped handing unfinished clerking to colleagues and remained at work for several hours beyond the end of their shift:

*“Most nights I leave between six and eight… [but I’m] paid until half four.” (Resourceful*,* T3)*.

### Access Work as Ongoing Labour

Disability access involved continual labour for which participants felt primary responsibility. Negotiating support required anticipation, explanation, and repeated advocacy.

Preparation began before employment. At T1, Alva expected to discuss ADHD with their educational supervisor because they might need support with *“organizing meetings”* and *“staying on top of my portfolio”.* What that support would involve remained unclear:

*“I don’t know how much support there will be… I surely won’t be the first to need that sort of support.” (Alva*,* T1).*

At T2, the hospital had proactively arranged a meeting with foundation tutors after reviewing Alva’s Supporting Trainees Entering Practice form, which recorded a specific learning difficulty. This gave Alva a known route back to disability support:

*“It’s nice that… you’re aware of me. And therefore*,* if I did need support*,* you would… already know who I am.” (Alva*,* T2)*.

Whilst this early contact reduced some uncertainty, it did not mean that F1 was compatible with all existing required support. For example, Alva described weekly treatment as *“impossible”* to schedule. Other participants could not identify a clear route to disability support like Alva had and had to identify responsible organisations themselves. Resourceful undertook substantial individual work to establish their rights during the transfer from UME to PGME, following a complete lack of guidance and support:

“*I then did the Googling and [found]… a firm… that specialises in disability-related employment matters.” (Resourceful*,* T2)*.

Resourceful’s account showed how access work developed across time. At T1, they could not know what shifts or nights would do to their health and therefore did not know what adjustments to request. By T2, this uncertainty had become a concrete breakdown between medical school and postgraduate support services. Resourceful had to establish the relevant legal responsibilities and coordinate communication between organisations. By T3, adjustments had been obtained, but their visibility prompted colleagues to question whether Resourceful’s health conditions warranted them, and difficult responses to handing over work contributed to Resourceful remaining at work beyond the end of their shifts.

Agreed disability support did not transfer consistently between placements. Participants repeatedly supplied information and pursued implementation after adjustments had been approved. Visible adaptations could prompt peer scrutiny, so access work also included managing colleagues’ responses. Near the end of F1, Victoria was initially allocated back to a surgical environment about which she had already raised repeated wheelchair-access concerns. The allocation changed only after she contacted previous supervisors and senior staff:

*‘I’d made so many complaints about the department being so inaccessible… [then] it was like*,* “Actually… you won’t be going to General Surgery now. You’ll be going somewhere else.“’ (Victoria*,* T4)*.

### Seeking Survivable Futures

Participants entered F1 with career aspirations already shaped by their disabilities. Participants considered whether different working patterns and clinical environments were compatible with their bodies, cognition, and ongoing support needs.

For Alva, survivability concerned whether clinical practice could be made compatible with established health support and life outside medicine. Before starting F1, the effect of night shifts on their medication was a source of uncertainty:

*“A big thing is how medication is going to work on nights… I’ve got no idea how that’s going to interact with me being up all night.” (Alva*,* T1)*.

By T2, Alva had adjusted the timing of their medication around night shifts:

*“Medication’s been fine*,* so I took my meds on my nights. I… switched taking them overnight… and that worked really well.” (Alva*,* T2).*

However, other support was harder to sustain, with weekly therapy becoming ‘*impossible’* to schedule. The organisation of their clinical work remained fixed, leaving Alva to fit management of their disability around their clinical time. Alva’s account demonstrates how successful participation in clinical practice often depends on the disabled doctor taking responsibility for reconciling fixed working patterns with their ongoing health needs.

Jenny compared specialties according to how their organisation affected her auditory processing. At T1, she had described several possible careers. After experiencing geriatric medicine, she used taster weeks in ENT and radiology to compare environments. She noticed *“less verbal elements”* in ENT ward rounds and *“less element of MDT [Multi-Disciplinary Team] conversation”* in radiology *(Jenny*,* T3).* She was uncertain whether she preferred procedural work in itself, or whether its lower auditory-processing demands made it feel more attractive:

*“I don’t know whether [it is] because… my brain is not very good at hearing [that is] making me hate the job*,* or it’s just myself… prefer[ring] procedural stuff more.” (Jenny*,* T3)*.

Other people’s views of Resourceful’s body also shaped which careers appeared available to them. At T2, they described enjoying emergency medicine and anaesthetics but recalled consultants advising that anaesthetics would be *“a more gentle specialty”* because of their *“physiological reserve” (Resourceful*,* T2*). By T3, after physically demanding surgical work and delays in receiving disability adjustments, Resourceful’s key career decision had shifted from specialty choice towards whether continuing in medicine was possible at all:

*“Things have been awful. Like I… have considered strongly and talked with friends about leaving.” (Resourceful*,* T3)*.

Across later interviews, participants increasingly evaluated roles and specialties in terms of sustainability, pace and whether they could realistically imagine themselves continuing long term. These aspirations were, mostly, still provisional at this early stage. Elise reflected on approaching future career decisions with uncertainty but increasing clarity:

*“I’m hoping once I get there*,* I’ll either know for certain… this environment is not going to work… or it might just work.” (Elise*,* T2)*.

Victoria’s later interviews showed how physical access could make one part of a specialty more accessible and survivable than another. For example, a community job involved more seated work than surgery:

*“Because a lot of my job is… sat down desk space… I’ve been able to use… my walker a bit more… because I then have more time to sit and rest.” (Victoria*,* T4)*.

These differences influenced how participants imagined their futures. Victoria was rethinking a longstanding interest in paediatrics in practical terms, balancing her interest with realism about training demands:

*“Whether I do pure Paeds*,* I don’t know*,* because I know the training pathway is awful… it’s rough.” (Victoria*,* T4)*.

However, rather than abandoning interests entirely, she explored alternatives that might be more sustainable:

*“I think well*,* if I did go down the route of like GP*,* you could always do special interest in Paediatrics…” (Victoria*,* T4)*.

Across accounts, success was increasingly defined as identifying contexts where practice felt possible without constant negotiation or self-justification. Participants moved toward self-defined forms of legitimacy grounded in fit, sustainability, and values. Notably, however, only two participants completed final interviews. Several participants who had described significant earlier challenges did not continue to the final timepoint. Accounts of consolidation and future planning may, therefore, reflect the experiences of those who remained engaged, rather than a universal trajectory. Nevertheless, later interviews suggest that repeated experiences of mismatch and adaptation shaped understandings of a survivable professional future.

## Discussion

### Summary of Findings

Our findings suggest that transition for disabled doctors is recurrent and context dependent. Participants entered F1 with prior experience of negotiating disability and access. The prospective design of our study has enabled us to demonstrate how their expectations were subsequently confirmed, modified, or contradicted. Familiar teams and workable task structures sometimes reduced difficulty, although positive experiences and supports were not always retained when rotations changed. Career plans also became more specific as participants experienced different working environments. The longitudinal contribution of this work is, therefore, an account of processes of transition that recurred and changed across clinical contexts for new disabled doctors.

It is important to recognise that many of the experiences described by participants, including those relating to uncertainty, changing team relationships, high workload, and fluctuating confidence, are characteristic of transition to practice more broadly [1, 4–6]. However, our findings suggest these challenges were intensified by the repeated negotiation of disability legitimacy and access needs across clinical contexts. Rotations required participants to re-establish adjustments and make new decisions about disclosure. The normative pace of much clinical work could conflict with auditory processing, fatigue, or mobility needs. Visible adjustments or altered working hours could also prompt colleagues to question whether support was justified. These processes added work to the transition already experienced by new doctors.

### Connections to Wider Research

This continued negotiation of professional and disability legitimacy extends research on legitimisation among disabled students in UME [35]. Our findings suggest that this demand to prove both professional and disability legitimacy continues into PGME, recurring across each new placement and clinical team. Jain’s capability imperative [36] helps explain this relationship. Medicine’s expectations concerning how doctors should work compel disabled learners to demonstrate that they meet particular bodymind standards. Our findings suggest that this imperative continues into PGME through placement changes, local decisions about adjustments, and expectations concerning pace. Jain’s account of disability legibility [22] is also relevant because being recognised as disabled could provide access to support, while exposing participants to additional scrutiny.

Seen through crip theory, our findings begin to illuminate ways in which disabled doctors work to “crip” the transition to PGME. Crip theory highlights overt resistance and system transformation, as well as subtler practices through which able-bodied norms are exposed and reworked. McRuer’s [16] concept of compulsory able-bodiedness spotlights how professional norms demand particular capacities, while Sandahl’s [37] notion of “spinning” shows cripping reveals these assumptions by working within and around them. From this perspective, cripping may take the form of strategic disclosure, selective compliance, refusal, or redefining what counts as successful participation in PGME. Our findings suggest that disabled doctors’ transition to practice involves precisely these kinds of practices, which engage with and unsettle dominant expectations.

Existing transition research conceptualises change as multidimensional and non-linear. Gordon et al.’s Transition to Trained Doctor model describes interacting transitions across personal, professional and organisational domains, with positive and negative experiences co-existing through time [1]. Our findings are consistent with this account, while extending this literature by showing how instability is reproduced through training structures and workplace norms. Rotational placements, variable expectations of pace and productivity, and locally negotiated access arrangements meant that relationships, credibility, and support were repeatedly re-established across contexts. Viewed through a crip lens, these practices show how participants negotiated compulsory able-bodiedness through selective disclosure, challenges to inaccessible allocations, and career decisions organised around sustainability rather than conformity to an idealised training pathway.

The distinction made by Jain and Scott between helping individuals to surmount barriers and changing the conditions that create them is useful when considering our findings [13]. Much of the support described by participants enabled them to manage a particular difficulty without changing the organisation of training. This is consistent with wider research documenting variable disability policies, fragmented accommodation processes, and difficulties accessing agreed support across UME and PGME [18, 24, 25, 27]. An adjustment could be formally approved but inconsistently implemented within a working environment, meaning participants had to advocate for support, repeatedly explain their needs, and develop knowledge of complex support systems. Our data show how accommodation processes can individualise access labour even when an organisation formally recognises its responsibility to provide support. The administrative and relational work required to make an adjustment functional was often transferred to disabled doctors themselves and undertaken alongside clinical learning and work. In addition, several participants also had to manage colleagues’ responses and defend the legitimacy of their support once it became apparent within a team. Because these processes could begin again after each rotation, formal adjustments did not necessarily produce stable access across doctors’ first year.

### Implications for Postgraduate Medical Education

These findings have implications for how transition support for disabled doctors is designed and delivered within PGME. Existing U.S. guidance identifies practical strategies for supporting disabled learners during the UME to PGME transition [28] and provides programme-level tools to support individuals in responding to needs [26]. While these U.S.-based strategies provide important starting points, our findings suggest that support cannot focus solely on helping individuals navigate existing systems. Our study participants described instability arising not only from disclosure decisions or access to accommodations, but from the repeated need to renegotiate legitimacy, pace, and support across clinical contexts. This suggests that support efforts must also address the organisational conditions that repeatedly reproduce these negotiations. From a crip perspective, we must recognise how everyday practices reveal the limits of current systems: rethinking norms of pace, productivity, and independence, rather than expecting individuals to continually adapt to them.

Addressing these dynamics requires approaches that move beyond individually negotiated accommodations toward more systemic forms of support. First, adjustments and access arrangements should be designed to travel more reliably across placements. Participants in this study repeatedly re-established adjustments and explained their needs to new teams, suggesting that mechanisms which allow agreed access arrangements to follow doctors across rotations may reduce the labour associated with maintaining access. Second, educational and clinical supervisors may benefit from clearer guidance on how to support disabled doctors during transitions between placements, including recognising how judgements about competence and legitimacy can become intertwined when adjustments are visible. Third, attention to workplace pace and workload expectations may be important when considering how disabled doctors experience early clinical practice. Participants described pace not only as workload but as a social signal of competence, indicating that efforts to improve transition experiences may also require reflection on how productivity and speed are culturally reinforced within clinical teams.

### Limitations

This intentionally small phenomenological sample enabled detailed engagement with six participants across eighteen interviews but cannot represent the full range of disabled doctors or support condition-specific conclusions. Participants also self-selected after recruitment through professional social-media networks. This recruitment route may have preferentially reached doctors who used these professional networks and were willing to disclose disability to researchers; their experiences may differ from doctors who do not use these networks or do not identify with disability as a label.

Attrition is a recognised challenge in longitudinal qualitative research [31], and the extent of attrition in this study constrained the later stages of our within-person analysis. All six participants contributed T1 and T2 interviews, four contributed T3 interviews, and two completed all four timepoints. The reflexive thematic analysis included all 18 interviews. However, within-person change beyond the initial transition could be examined for four participants, and complete accounts approaching the end of F1 were available for only two. Later accounts of career planning and sustainability should therefore be interpreted cautiously, as they may disproportionately reflect participants who remained able or willing to continue engagement. Participants were not required to provide reasons for discontinuing, so we cannot determine whether attrition was related to the experiences described. This limits claims about experiences approaching the end of F1, while comparisons between T1 and T2 draw on the full sample.

The heterogeneity of participants’ disabilities also affects how these findings may be transferred to other contexts. The study supports transferability at the level of the disability-related processes described, including repeated access work and the negotiation of legitimacy within rotational training. It cannot establish how these processes are experienced within particular disability groups or whether they occur across the wider population of disabled doctors. We have provided contextual detail to allow readers to judge the relevance of the findings to other PGME settings.

## Conclusion

Our study extends existing transition literature by demonstrating how organisational arrangements within PGME may repeatedly reproduce negotiations of legitimacy and access for disabled doctors throughout the first postgraduate year. Our findings suggest that transition should be understood as a structural phenomenon shaped by workplace norms and training design. Recognising and addressing these dynamics is an important step toward creating training environments in which disabled doctors can participate in clinical practice equitably.

## Supplementary Information

Below is the link to the electronic supplementary material.

Supplementary Material 1 (DOCX 18.4 KB)

Supplementary Material 2 (DOCX 44.3 KB)

## Data Availability

The datasets generated during and/or analysed during the current study are available from the corresponding author on reasonable request.

## Footnotes

1 For more information, please see: https://foundationprogramme.nhs.uk/.

## References

[1] Gordon L, Teunissen PW, Jindal-Snape D, Bates J, Rees CE, Westerman M, Sinha R, van Dijk A. An international study of trainee-trained transitions: Introducing the transition-to-trained-doctor (T3D) model. Med Teach. 2020;42(6):679–88. 10.1080/0142159X.2020.1733508 32150488

[2] Grassi L, McFarland D, Riba MB. The Problem of Burnout, Depression, and Suicide in Physicians: A General Overview. Depression, Burnout and Suicide in Physicians: Insights from Oncology and Other Medical Professions. 2021 Dec 1:1 – 0.

[3] Hickam DH, Severance S, Feldstein A, Ray L, Gorman P, Schuldheis S, Hersh WR, Krages KP, Helfand M. The effect of health care working conditions on patient safety. Evidence report/technology assessment (Summary). Agency for Healthcare Research and Quality (US). 2003;1(74):1–3. https://www.ncbi.nlm.nih.gov/books/NBK36875/. PMC4781355 12723164

[4] Illing JC, Morrow GM, Rothwell nee Kergon CR, Burford BC, Baldauf BK, Davies CL, Peile EB, Spencer JA, Johnson N, Allen M, Morrison J. Perceptions of UK medical graduates’ preparedness for practice: a multi-centre qualitative study reflecting the importance of learning on the job. BMC Med Educ. 2013;13(1):34. 10.1186/1472-6920-13-34 PMC3599362 23446055

[5] Teunissen PW, Westerman M. Junior doctors caught in the clash: the transition from learning to working explored. Med Educ. 2011;45(10):968–970. 10.1111/j.1365-2923.2011.04052.x 21916936

[6] Lundin RM, Bashir K, Bullock A, Kostov CE, Mattick KL, Rees CE, Monrouxe LV. I’d been like freaking out the whole night: exploring emotion regulation based on junior doctors’ narratives. Adv Health Sci Educ. 2018;23(1):7–28. 10.1007/s10459-017-9769-y PMC5801373 28315113

[7] Riley R, Buszewicz M, Kokab F, Teoh K, Gopfert A, Taylor AK, Van Hove M, Martin J, Appleby L, Chew-Graham C. Sources of work-related psychological distress experienced by UK-wide foundation and junior doctors: a qualitative study. BMJ open. 2021;11(6):e043521. 10.1136/bmjopen-2020-043521 PMC8231022 34162634

[8] Keenan J, Doherty M, Shaw SC. The experiences of autistic doctors transitioning into clinical practice in the UK: a phenomenological study. Teaching and Learning in Medicine. Dec. 2025;19:1–5. 10.1080/10401334.2025.2608185 41530981

[9] Atherley A, Dolmans D, Hu W, Hegazi I, Alexander S, Teunissen PW. Beyond the struggles: a scoping review on the transition to undergraduate clinical training. Med Educ. 2019;53(6):559–70. 10.1111/medu.13883 PMC6593677 31012141

[10] Frost HD, Regehr G. I AM a Doctor Negotiating the Discourses of Standardization and Diversity in Professional Identity Construction. Acad Med. 2013;88(10):1570–7. 10.1097/ACM.0b013e3182a34b05 23969361

[11] Volpe RL, Hopkins M, Haidet P, Wolpaw DR, Adams NE. Is research on professional identity formation biased? Early insights from a scoping review and metasynthesis. Med Educ. 2019;53(2):119–32. 10.1111/medu.13781 30656747

[12] Wyatt TR, Balmer D, Rockich-Winston N, Chow CJ, Richards J, Zaidi Z. Whispers and shadows’: A critical review of the professional identity literature with respect to minority physicians. Med Educ. 2021;55(2):148–58. 10.1111/medu.14295 33448459

[13] Jain NR, Scott I. When I say… removing barriers. Med Educ. 2023;57(6):514–5. 10.1111/medu.15075 36869431

[14] Cousins N. Internship: preparation or hazing? JAMA. 1981;245(4):377. 6109029

[15] Mahood SC. Medical education: Beware the hidden curriculum. Can Fam Physician. 2011;57(9):983–5. PMC3173411 21918135

[16] McRuer R. Crip theory: Cultural signs of queerness and disability. New York, NY: New York University; 2006.

[17] Mitchell D, Snyder S, Ware L. [Every] Child Left Behind Curricular Cripistemologies and the Crip/Queer Art of Failure. J Literary Cult Disabil Stud. 2014;8(3):295–314.

[18] Brown ME, Burford B, Vance G. Barriers, facilitators and solutions to equitable career progression for disabled doctors: an integrative review. BMJ open. 2025;15(10):e106639. 10.1136/bmjopen-2025-106639 PMC12496058 41043846

[19] Maggio LA, Brown KR, Costello JA, Konopasky A, Bidny D, Konopasky A. Disability in undergraduate medical education in the United States: A scoping review. Acad Med. 2025;100(Supplement2):S64–73. 10.1097/ACM.0000000000006154 40577649

[20] Meeks LM, Foreword. Disability Inclusion in Undergraduate Medical Education. Acad Med. 2025;100(Supplement2):S49–53. 10.1097/ACM.0000000000006151 40550203

[21] Makri I. Doctors’ wellbeing and experience of workplace transitions [doctoral dissertation]. Liverpool, UK: University of Liverpool; 2020.

[22] Jain NR. Legibility: knowing disability in medical education inclusion. Adv Health Sci Educ. 2024;29(2):507–30. 10.1007/s10459-023-10268-1 PMC11078834 37479819

[23] Shrewsbury D. Disability and participation in the professions: examples from higher and medical education. Disabil Soc. 2015;30(1):87–100.

[24] Meeks LM, Taylor N, Case B, Stergiopoulos E, Zazove P, Graves L, McKee M, Swenor BK, Salgat A, Cerilli C, Joshi H. The unexamined diversity: disability policies and practices in US graduate medical education programs. J graduate Med Educ. 2020;12(5):615–9. 10.4300/JGME-D-19-00940.1 PMC7594787 33149832

[25] Meeks L, Case B, Joshi H, Harper D, Graves L. Disability policies and practices in family medicine residencies: a CERA study. Fam Med. 2021;53(3):211–4. 10.22454/FamMed.2021.726731 33723820

[26] Salinger M, Sheets ZC, Bienstock JL, Rudkowski JC, Shaw KR, Edje L, Messman A, Fisher H, Fousone M, Kakara M, Martin K. The Disability Policy Toolkit: Resource Development and Applications Within Graduate Medical Education. J graduate Med Educ. 2025;17(6):792–7. 10.4300/JGME-D-25-00887.1 PMC12710346 41415981

[27] Moreland CJ, Pereira-Lima K, Lee KT, Viglianti EM, DeAndrea-Lazarus IA, Zeveney A, Meeks LM. Disability accommodation access and requests in US internal medicine residents with disabilities. JAMA Netw Open. 2026;9(3):e263392. 10.1001/jamanetworkopen.2026.3392 PMC13036577 41910974

[28] Sheets ZC, Fausone M, Messman A, Ortega P, Ramsay J, Creasman M, Charnsangavej N. Preparing to thrive: Supporting learners with disabilities through the undergraduate-to-graduate medical education transition. Acad Med. 2025;100(Supplement2):S161–5. 10.1097/ACM.0000000000006136 40550105

[29] Byrne MH, Brown ME. Developing a Community of Scholars for Students, Residents, and Early-Career Researchers: MedEd Collaborative—A Health Professions Education Research Collaborative. Acad Med. 2025;100(8):919–24. 10.1097/ACM.0000000000006040 PMC12321344 40737217

[30] Neubauer BE, Witkop CT, Varpio L. How phenomenology can help us learn from the experiences of others. Perspect Med Educ. 2019;8(2):90–7. 10.1007/s40037-019-0509-2 PMC6468135 30953335

[31] Gordon LJ, Shaffer K, Finn GM, Lamb S, Brown ME, Chow C. The Unique Possibilities of Longitudinal Qualitative Research in Health Professions Education. Clin Teach. 2025;22(4):e70126. 10.1111/tct.70126 40542582

[32] Jain NR, Varpio L. Designing for justice: How universal design theory could bolster health professional education research. Focus Health Prof Education: Multi-Professional J. 2023;24(4):136–50.

[33] Heaton J. * Pseudonyms are used throughout: A footnote, unpacked. Qualitative Inq. 2022;28(1):123–32.

[34] Braun V, Clarke V. Thematic analysis: a practical guide. London: Sage; 2021.

[35] Easterbrook A, Bulk L, Ghanouni P, Lee M, Opini B, Roberts E, Parhar G, Jarus T. The legitimization process of students with disabilities in health and human service educational programs in Canada. Disabil Soc. 2015;30(10):1505–20.

[36] Jain NR. The capability imperative: theorizing ableism in medical education. Soc Sci Med. 2022;315:115549. 10.1016/j.socscimed.2022.115549 36413858

[37] Sandahl C. Queering the crip or cripping the queer? Intersections of queer and crip identities in solo autobiographical performance. GLQ: J lesbian gay Stud. 2003;9(1):25–56.

