# Supplementary material for: Cripping the Transition to Practice: A Longitudinal Qualitative Analysis of Disabled Doctors’ First Year Experiences
Source: Med Sci Educ. 2026 Sep 5;36(6):3141–51. doi: 10.1007/s40670-026-02873-7 (PMC13759751; doi:10.1007/s40670-026-02873-7)
Supplement: Supplementary file 1 [file 40670_2026_2873_MOESM1_ESM.docx]

# Supplementary material

Interview guide

**INTERVIEW 1**

1. Can you tell us a little about yourself and your journey to where you are today?
2. Can you tell us about your experiences as a disabled medical student?
   a. Moments of joy and thriving
   b. Difficulties and barriers
3. Have you been supported as a medical student to participate fully in all activities of learning and assessment?
   a. If so, how?
   b. If not, are you able to tell us a little more about this?
   c. May be relevant : whether or not they have chosen to disclose disability to their medical school / some people in the school and why/why not
4. Are there any particular resources, support systems, or networks that have been invaluable to you as a medical student so far?
5. How are you feeling about becoming a doctor in the near future?
   a. Can you tell us more about why you are feeling that way?
   b. Any hopes for when you become a doctor?
   c. Any concerns about becoming a doctor?
6. Have you disclosed your disability/disabilities to your future medical employer?
   a. Can you tell us more about this decision?
   b. Do you have any accommodations in place at this point? Why/why not? If yes – what are these accommodations, and how do you envision them supporting once in work.
7. Are there any changes or improvements you would like to see to better support disabled medical students, particularly thinking of this moment in time as you prepare to become a doctor?
8. We would like to use names for our participants, such as yourself, rather than numbers when we tell the story of our data. It would be wonderful if you were willing to choose a name for your stories, not your own name, but something you would be happy to see yourself represented by. Can you think of a name you would be happy for us to use?
9. Any other questions/anything you want to add?

**INTERVIEW 2**

Welcome participant back, thank them again for their time.

1. How have things been, since we last spoke?

[Insert specific questions based on interview 1 here]

General questions for all participants (if covered by specific questions above, no need to repeat):

2. Have there been any significant moments or experiences in your transition to practicing as a doctor that stood out to you?
3. Reflecting on your initial period of working as a doctor, how have you navigated challenges related to your disability? Are there new strategies or supports you've found helpful?
4. In what ways, if any, has your perspective on your role as a disabled doctor evolved since we last spoke?
5. Have there been any changes or updates regarding accommodations or support systems at your workplace since our last discussion?
6. Can you describe any specific instances where you felt your identity as a disabled doctor was particularly salient or came into play in a new way?
7. Is there anything you wish your colleagues or the healthcare system at large understood better about working with disabled doctors?

**INTERVIEW 3**

Welcome participant back, thank them again for their time.

1. How have things been, since we last spoke?

[Insert specific questions based on interview 2 here]

General questions for all participants (if covered by specific questions above, no need to repeat):

1. Now you are X amount of time [change depending on date of interview] into work as a doctor, do you have any reflections on your journey to date?
2. Can you share any specific experiences or moments that have been particularly significant to you since we last spoke?
3. Have you encountered any new challenges or barriers related to your disability?
   1. How have you and your workplace responded to these?
4. Reflecting on the ongoing adjustments and accommodations for your disability, how have these impacted your daily work and interactions with colleagues and patients?
   1. Are there any additional accommodations or support systems you’ve found to be necessary or particularly helpful at this stage in your career?
   2. Anything you need, that you don’t have access to?
5. Can you describe any specific instances where you felt your identity as a disabled doctor was particularly salient or came into play in a new way?

**INTERVIEW 4:**

Welcome participant back, thank them again for their time.

1. How have things been, since we last spoke?

[Insert specific questions based on interview 3 here]

General questions for all participants (if covered by specific questions above, no need to repeat):

1. Looking back over your first year as a doctor, what stands out most?
2. What’s been most unexpected or surprising about FY1?
3. Can you tell me about a moment you’re proud of from this year?
4. What’s been hardest? (emotionally, physically, professionally)
5. If you could give advice to yourself a year ago, what would it be?
6. If your team or supervisors could know one thing to better support you, what would it be?
7. What’s the latest in your FY2 planning, and beyond, for your career?
8. This is our last interview, do you have anything you would like to add on your experience across this research project, that you feel we haven’t had chance to cover or consider?
