# Supplementary material for: Cripping the Transition to Practice: A Longitudinal Qualitative Analysis of Disabled Doctors’ First Year Experiences
Source: Med Sci Educ. 2026 Sep 5;36(6):3141–51. doi: 10.1007/s40670-026-02873-7 (PMC13759751; doi:10.1007/s40670-026-02873-7)
Supplement: Supplementary file 2 [file 40670_2026_2873_MOESM2_ESM.docx]

| **Editor/reviewer comment** | **Response and changes made** |
| --- | --- |
| **Associate Editor** |  |
| This is an interesting and potentially important qualitative study addressing the experiences of disabled doctors during the transition to clinical practice. The longitudinal design, phenomenological approach, reflexive thematic analysis, positionality statement, and use of an interview guide are strengths. However, substantial revisions are needed before the manuscript can be considered further.  Overall, the manuscript has merit and addresses an important topic, but significant revisions are required to strengthen methodological transparency, longitudinal analysis, interpretation, and relevance to medical education. | Thank you for recognising the importance of this topic and the strengths of our longitudinal design, phenomenological approach, reflexive thematic analysis, positionality statement and interview guide. We have addressed the requested revisions point by point below and hope you will agree that the manuscript is now stronger. |
| Sample and sampling: The sample of six participants is small and highly heterogeneous, given the broad definition of disability used. The authors should explain why recruitment stopped at six participants and clearly acknowledge the limited transferability of the findings. Greater detail about participants' disabilities and relevant demographics should be provided, preferably in a table while maintaining confidentiality. The implications of recruiting participants who publicly discuss disability on social media should also be addressed as a major limitation. | We agree that the rationale for the sample and its limits required clearer reporting. Recruitment was prospectively time-bound because participants had to enter before commencing F1 so that the study could follow a common transition point. Six eligible participants enrolled during that window. The study uses a phenomenological approach which requires a small sample – we are not aiming in this study for statistical representativeness or coverage of all disability experiences. We have made this explicit in the revisions to the paper and expanded the aggregate description of disability experiences. We have chosen not to add a participant-level demographic table because combinations of disability, training stage and public social-media recruitment would materially increase the risk of deductive identification in a six-person sample. This would risk breaching our confidentiality arrangements with participants and also our institutional ethical approval. We have instead reported characteristics in aggregate and strengthened the limitations section concerning the recruitment route and transferability. |
| Longitudinal analysis: Only two participants completed the study through the final interview. This needs to be clearly stated in the abstract and discussed as an important limitation. The authors should explain participant attrition and provide a stronger analysis of how experiences and narratives evolved throughout the first year. | We agree. The abstract now reports participation at each stage: six participants completed T1 and T2, four completed T3, and two completed T4, producing 18 interviews. Participants were invited to subsequent interviews and flexible formats remained available, but they were not required to give reasons for discontinuing (in line with our ethics board recommendations); we therefore cannot attribute attrition to any particular cause. We have substantially revised the Results to label our quotations by timepoint and show within-person comparison where relevant. We hope you agree that the revised analysis does not imply that all participants followed a shared developmental sequence, this was not our intention. We have tried to make changes to identify context-dependent change, including improvements that were lost after rotation and career plans that were refined throughout the course of experience during the first year of practice, rather than newly formed at T4. |
| Methods and analysis: The reflexive thematic analysis should be described more explicitly, including the analytic framework and appropriate methodological references (e.g., Braun and Clarke, if applicable). Author roles in interviewing, coding, and analysis should be clarified, and unexplained acronyms should be avoided. | Our phenomenological orientation guided attention to lived experience and meaning-making, reflexive thematic analysis provided us with a flexible analytic method, and crip theory served as a sensitising interpretive framework. The Methods now describes Braun and Clarke's recursive phases and the subsequent comparison of individual participants' earlier and later interviews. Author roles in interviewing, transcript production, coding, analysis and wider team discussion are stated directly. Acronyms are introduced before use. |
| Results and Discussion: The Results would benefit from a participant characteristics table and a clearer presentation of themes and representative quotations. The Discussion currently repeats portions of the Results and should provide more interpretation. In particular, the authors should distinguish experiences common to all new physicians from those specifically shaped or intensified by disability and connect the findings more explicitly to crip theory and existing literature. | We revised the Results around timepoint-labelled quotations and added a table summarizing the themes for additional clarity. We chose not to move quotations into a separate table because their relationship to the analytic narrative, and situation within this, is important for context. The Discussion has been rewritten to reduce summary and focus on interpretation. It now distinguishes ordinary transition demands from disability-specific access labour and interactions between pace, impairment, adjustments and judgements of competence. Crip theory is applied to concrete practices in the data, including selective disclosure, challenging inaccessible allocations, refusing individualised responsibility and redefining successful participation through sustainability. |
| Relevance to medical education: One reviewer questioned the fit with Medical Science Educator. The authors should strengthen the manuscript's explicit contribution to medical education and PGME, including implications for accommodations, assessment, supervision, clinical learning environments, and transition from undergraduate to postgraduate training. | We agree and have made the educational contribution explicit. The revised Discussion includes a dedicated subsection on implications for PGME. It addresses transfer and implementation of adjustments, named responsibility before placement, separation of competence judgements from assumptions about disability or altered hours, consistency in assessment and progression guidance, and review of workload, information processing and physical access within clinical learning environments. |
| **Reviewer 1** |  |
| Given the broad definition of disabled learners and doctors, it seems unlikely that a sample of 6 individuals can be considered representative of the lived experiences of such a diverse group. This limitation and constraint should be presented to the reader upfront and at all levels of the paper. | We agree that the study cannot represent the full diversity of disabled doctors, but respectfully distinguish transferability in qualitative phenomenological research from statistical representativeness. The intentionally small sample supported repeated, in-depth engagement across 18 interviews. We have removed any implication of comprehensive coverage, stated that the sample was not intended to represent all disabled doctors, and narrowed claims to the processes described in this sample and context. We have also clarified in the discussion that transferability should be considered at the level of the disability-related processes identified within this cohort, rather than as evidence that all disabled doctors or disability groups share these experiences. We now state that the small cross-disability sample cannot support condition-specific conclusions and provide contextual information we hope will allow readers to judge relevance to other PGME settings. |
| The materials and methods in the abstract do not make clear that only two participants were followed until the end of the study. | The Methods portion of the abstract now reports completion at all four timepoints. |
| Pg 3. Ln 8. I believe the authors meant to state "all shifting simultaneously".  Pg 4. Ln 1. "studies offer". | The wording has been checked and corrected. We also edited the surrounding sentences for clarity. |
| Pg 6. Ln 38-39. Seems "depth" is repeated.  Pg. 6, Ln. 39. The word "myriad" is commonly understood to mean a very large number. This is causing cognitive dissonance in me with a sample of 6. I recommend changing for "several" or "diverse". | We removed the duplicated word and replaced the broad wording with a specific aggregate description of disability experiences. |
| A table or list of the disabilities represented by the participants would be helpful to better understand the coverage of perspectives in the data. | We have added a more specific aggregate list of disability experiences, including physical and mobility impairments, sensory and auditory-processing disabilities, dyslexia, ADHD, chronic illness and mental health conditions. We have not linked these characteristics to individual pseudonyms because doing so would increase the risk of identification in this small publicly recruited sample and doing so would breach our ethical approval. |
| Although there is no sample size agreed for phenomenological studies, the broad scope of this study (physicians with disabilities) makes it hard to believe that six perspectives can lead to a holistic understanding of the phenomenon. I would like to see at least some discussion regarding this. | We agree that participants’ disabilities and access needs were heterogeneous. This was a cross-disability study of how disability was experienced and negotiated during the transition to clinical practice. It did not investigate the experience of a particular impairment or diagnostic group. The analytic coherence of the sample came from participants’ shared experience of entering F1 as disabled doctors within the same UK postgraduate training structure. We did not assume that different disabilities produced homogeneous or equivalent experiences. The analysis examined disability-related processes identified across accounts, including the negotiation of disability legitimacy, access labour, interactions between impairment and workplace pace, and the development of sustainable forms of participation. We retained variation in how participants experienced these processes according to their impairments, access needs and clinical environments. The heterogeneity of disability within the sample therefore supported a cross-disability analysis of how common PGME structures could produce different forms of disablement.  However, we do appreciate and agree that six participants cannot capture the full range of disabled doctors’ experiences or support conclusions about particular disability groups. We have made these limits explicit within the revised manuscript, particularly within the limitations section of the discussion. |
| **Reviewer 2** |  |
| GENERAL: The introduction/background is well organized and provides information suitable for a novice in this field. However, the rationale for the research appears simplistic and based on common sense rather than a genuine research question. A few compound sentences were confusing, and I had to read them a few times to clarify. | We agree that the previous rationale did not define the empirical and conceptual gap sufficiently. We have revised the Introduction to distinguish our study from existing transition and disability research. Existing work documents preparedness and adjustment during medical transitions, while disability research identifies barriers and accommodation difficulties within UME and PGME. However, prospective evidence following disabled doctors from before entry to practice across their first postgraduate year remains limited.  The revised Introduction explains why longitudinal evidence is required. Repeated interviews allow comparison between concerns anticipated before work and experiences subsequently encountered, reduced or reinterpreted across placements. This helps distinguish difficulties associated with learning a new professional role from those produced through interactions between disability, changing clinical environments and the local implementation of access arrangements. We also position the study in relation to recent single-timepoint phenomenological research with autistic doctors and existing UME to PGME guidance. |
| Abstract section, lines 46 and 48: I'm not sure what you mean by "Support for disabled doctors during transition must address the structural conditions that reproduce these demands." | We have replaced this with a concrete statement about transferring and implementing adjustments across placements and addressing how expectations concerning pace, productivity and independence influence supervision and competence judgements. |
| Page 3, line 31: remove the "s" on the word "toward". | Corrected. |
| The research design and methodology are sound. Given the small sample size, how many times did you contact participants to ensure they completed each interview? Having only a third participate in the final interview is concerning, particularly for a thematic analysis. | We agree that the extent of attrition requires transparent reporting. At each subsequent timepoint, participants received an initial email invitation and, if they did not respond, up to three email reminders. Flexible interview formats were also offered. Despite this follow-up, all six participants completed T1 and T2, four completed T3, and two completed T4. Participants were not required to provide reasons for discontinuing (as per our ethics committee requirements), so we cannot determine why individual participants did not continue.  The Abstract now reports completion at each timepoint, and the Methods describes the invitation and reminder process. The reflexive thematic analysis included all 18 interviews. Incomplete participation specifically constrained within-person comparisons beyond T2 and claims about experiences approaching the end of F1. We have made this explicit in the Limitations and interpreted later accounts of career planning and sustainability cautiously. |
| Since the authors performed a reflexive thematic analysis (as stated on Pages 7 and 8), did they use Braun's and Clarke's technique (https://www.tandfonline.com/doi/abs/10.1191/1478088706qp063oa)? If so, this citation should be included in the references and detailed in the methods section. | Yes. The manuscript already cited Braun and Clarke, but the analytic procedure was not described sufficiently. We now describe the six recursive phases and the longitudinal comparison undertaken after timepoint-level coding. We also clarify how RTA related to phenomenology and crip theory. |
| Page 6, line 27/28: The sentence "MELB/AHB provided…" seems like a new acronym that needs to be defined. To reduce confusion, change the sentence to read, "Two authors (MELB and AHB) provided all..."  Page 6, line 37: Sentences should not begin with a number or acronym unless they are spelled out. Change to read, "Six participants…"  Page 6, lines 37-38: This is confusing, "…on in-depth depth engagement…"  Page 7, lines 56-59: The sentence "MELB/AHB produced…" seems like several new acronyms that need to be defined. To reduce confusion, change the sentence to read, "The interviewing authors (MELB and AHB) produced verbatim... Both MELB and OH led the analysis, where AHB, GF, and NRJ supported coding…"  Page 8, line three: Change to, "dataset. Authors MELB and OH then…" | Agreed. We now use formulations such as 'Two authors (MELB and AHB)' and 'Six eligible participants enrolled', and clarify roles throughout the Methods. |
| Page 6, lines 57-59: Please remove the years, as these could be used to identify participants and should be removed following IRB/Ethics Board requirements. | We respectfully retained the study-level dates because they identify the cohort and period of data collection rather than an individual participant, and are important for transparent reporting of a prospective longitudinal study. We have, however, avoided linking demographic characteristics to individual pseudonyms and report potentially identifying characteristics only in aggregate. |
| Supplementary material, Interview #1: I think the word "student" is incorrect here: "Have you been supported at medical student to participate fully in all activities of learning and assessment?" What was this question? | Corrected to 'Have you been supported as a medical student...'. |
| GENERAL: Large portions of the Discussion seem to just repeat the Results without bringing in a thoughtful interpretation of the findings. | We agree that the distinction between reporting and interpretation could be stronger. We rewrote the Discussion to focus on the longitudinal contribution, relationships with existing transition and disability literature, the capability imperative, concrete application of crip theory and implications for PGME. We added subheadings for additional clarity and to improve readability. Repetition of individual results has been reduced. |
| Based on the Results section, the following statement may also apply to any new physician ("Rotational training structures repeatedly reset relationships, meaning that professional legitimacy had to be continually re-established even after participants felt accepted within a team.") How is it different for people with disabilities? Many of the participants' statements do not provide evidence that it is more difficult for them. Are there better examples to illustrate this? | We agree that rotation and changing relationships are common transition experiences. The revised Results and Discussion separate these from the disability-specific work occurring alongside them. Within-person examples now show how rotation required renewed implementation of adjustments, disclosure, management of others' assumptions and re-establishment of disability legitimacy. We also include Jenny's comparison between fast ward rounds and self-paced consultations, which shows how the same participant's difficulty changed with the learning environment. |
| Many of the results could be presented in a table that groups the statements into the themes you created to make them easier to read. | We added Table 2, which presents each theme and summarises each clearly. Representative quotations remain integrated with the analytic narrative because removing them into a separate table would weaken the connection between data and interpretation. |
| SPECIFIC: The results would be stronger if you included a table listing the codes you identified alongside the statements used for those codes. Consider adding details on how many times each code was identified, which would support the value of the code and how it supported a theme. | We respectfully did not add code-frequency counts. In Braun and Clarke’s approach to reflexive thematic analysis, themes are constructed as patterns of shared meaning in relation to the research questions; their analytic value is not established by the frequency of code application. Frequency counts would also imply a coding-reliability or content-analysis logic inconsistent with the stated method, and our interpretivist paradigm. We instead strengthened methodological transparency, added a theme table, labelled quotations by timepoint and provided within-person comparisons so readers can assess the relationship between interpretation and data. |
| Reference #16 (McRuer, R.) is in a different format; it is not in AMA format.  Reference #21 (Makri, I.) is in a different format; it is not in AMA format. | Corrected. |
| GENERAL: There were no tables or figures but including them would improve this paper. | The submitted manuscript included Table 1 reporting participation at each interview, although we recognise it may have been overlooked. We have retained Table 1 and added Table 2 to summarise each theme. No figure was added because the central relationships are presented more precisely in the table and narrative. |
| GENERAL: The conclusions do not seem to overstate findings.  GENERAL: These seem appropriate for the paper's topic, and about 50 percent of the references were within the last five years. | Thank you. We have retained the proportionate conclusion and the current literature base. |
| **Reviewer 3** |  |
| The research questions informed by crip theory are appropriate.  Qualitative methods, specifically the phenomenological approach and reflexive thematic analysis of individual interview data is appropriate.  The authors include a positionality statement and reflexivity, which is appropriate.  Including the interview guide as supplemental material is a strength to provide the reader with insight to interpret the interviews.  The authors provide example quotations to bolster their interpretation. | Thank you. We have retained these elements while clarifying their methodological relationship and reporting. |
| Six is a small sample size. Why did the authors stop with six participants? | Recruitment was bounded by the need to enrol final-year students before they commenced F1 so that data collection could begin at a common pre-transition point. Six eligible participants enrolled during this period. The sample size was also appropriate for the repeated, in-depth engagement sought through the study’s phenomenological orientation. These six participants contributed 18 interviews across the study. |
| Given diverse experiences and the heterogeneity of various disabilities, I have concerns about the transferability of a study with only 6 participants that could span self-identities including "disabled, neurodivergent, D/deaf, and/or as someone who experiences impairment, chronic illness, or mental health concerns." Can the authors provide more specifics on the demographics and which self-identified conditions the participants had? | We have expanded the aggregate description of disability experiences. We have not added participant-level cross-tabulation because combinations of condition, demographic information, training stage and public recruitment route would increase deductive identification risk. This decision is now explained within our manuscript. We have provided more information relating to the value of looking across various disabilities, rather at one specific condition, in response to reviewer 2, above. Please refer to the reviewer 2 response for more detail / justification of this approach. |
| The paper would be strengthened by the inclusion of a list of the specific disabilities or other demographic information of the participants. | Please see our response to the previous comment. We have added a more specific aggregate description of the disabilities represented but have not linked conditions or demographic characteristics to individual pseudonyms because this would increase deductive identification risk and conflict with protecting confidentiality of participants. |
| I am somewhat confused by the inclusion of names ("Victoria", "Jenny", etc.) in the results. Are these pseudonyms? Should the reader assume gender from the names? If not, why not use "participant 1", participant 2", etc. and include with all quotations, not just some? | All names used in the Results are pseudonyms. Three participants selected their own pseudonyms, while pseudonyms were generated for the remaining three participants to maintain a consistent approach. We retained pseudonyms rather than participant numbers because this respected the choices of participants who selected how they wished to be represented. The revised manuscript states that names should not be used to infer gender, and all quotations are labelled by pseudonym and interview timepoint. |
| Having longitudinal interviews is a strength of the study. However, there is limited analysis on how narratives evolved throughout the course of the study. I expected richer analysis with a longitudinal qualitative study. | We agree. The Results have been substantially revised around within-person comparison. Quotations are labelled by timepoint, Table 2 summarises change within each theme, and the narrative distinguishes within-person change from patterns constructed across the sample.  Resourceful’s account is followed from uncertainty about support needs at T1, through a breakdown in coordination between UME and PGME services at T2, to obtaining adjustments while experiencing peer scrutiny and extended working time at T3. Jenny’s account develops from anticipated difficulties with unfamiliar terminology at T1 to the effects of fast collective ward rounds at T3 and the reduced impact of her auditory-processing disability during self-paced consultations at T4. These comparisons demonstrate context-dependent change without implying that participants followed a common developmental sequence. |
| The limitations are appropriate. However, in my opinion, the authors underemphasize the impact of their sampling strategy. Not all individuals that identify as having a disability broadcast it on social media. Exclusively sampling from those that discuss their disability publicly on social media is a SUBSTANTIAL limitation of the study. | Recruitment advertisements were circulated publicly through professional and institutional social-media accounts. Participants were not required to have discussed their disability publicly on social media. Nevertheless, we agree that this recruitment route may have preferentially reached doctors who used these professional networks and were willing to disclose disability to researchers. The revised Limitations acknowledges that this may have shaped the experiences described and the forms of advocacy represented in the analysis. |
| Interesting paper. However, I am not sure it addresses the focus of this journal, medical science education. | We have made the educational contribution of the paper more explicit. The revised Discussion includes a dedicated subsection on implications for PGME. It addresses the transfer and implementation of agreed adjustments across rotations and the guidance required by educational and clinical supervisors. It also considers how competence judgements can become entangled with assumptions about disability or altered working hours, and how expectations concerning pace and workload affect participation within clinical learning environments. |
